# Proximal Hamstring Tendon Avulsions: A Survey of Orthopaedic Surgeons’ Current Practices in the Nordic Countries

**DOI:** 10.1186/s40798-022-00439-6

**Published:** 2022-04-11

**Authors:** Sofia Laszlo, Martin Nilsson, Elsa Pihl, Ville M. Mattila, Jörg Schilcher, Olof Sköldenberg, Frede Frihagen, Kenneth B. Jonsson

**Affiliations:** 1 Department of Surgical Sciences, Uppsala University, 751 85 Uppsala, Sweden; 2 Department of Orthopaedics and Traumatology, Östersund Hospital, Östersund, Sweden; 3 Karolinska Institutet, Department of Clinical Sciences, Danderyd Hospital, Unit of Orthopaedics, Stockholm, Sweden; 4 Department of Orthopaedics and Traumatology, Tampere University and University Hospital, Tampere, Finland; 5 Department of Orthopaedic Surgery and Department of Biomedical and Clinical Sciences, Linköping University, Linköping, Sweden; 6 Department of Orthopaedic Surgery, Østfold Hospital Trust, Grålum, Norway; 7 Institute of Clinical Medicine, University of Oslo, Oslo, Norway

**Keywords:** Hamstrings, Tendon injury, Surgical intervention

## Abstract

**Background and purpose:**

Evidence guiding the decision on whether to treat proximal hamstring tendon avulsions (PHA) operatively or non-operatively is very limited. The aim of this study was to identify the current practices and the rationale behind PHA treatment decisions in the Nordic countries.

**Methods:**

A survey was sent to orthopaedic surgeons in Sweden, Norway, Finland and Denmark. The study population consisted of responding surgeons with exposure to surgical treatment of PHA (*n* = 125). The questions covered surgeon and unit characteristics, and surgeons’ understanding of the evidence for treatment, and they explored which patient and injury factors influence treatment allocation.

**Results:**

Although some surgeons indicated a preference for one of the treatments, 84% stated that the treatment decision was based on patient and injury-related factors. Severe obesity, drug abuse, a sedentary lifestyle, age > 60 years and delayed diagnosis (> 6 weeks) were considered contraindications to surgical treatment. Also, there was agreement that patients expressing a preference for non-operative treatment should not be operated. Complete avulsions with tendon dislocation ≥ 2–3 cm on MRI were relative indications for surgical treatment.

The majority of surgeons did not believe that operatively treated patients did better than non-operatively treated patients and experienced that patients, generally, were satisfied with the treatment result, regardless of the type of treatment. Most surgeons had experienced significant complications to operative treatment.

**Conclusion:**

Current practices varied among different units, and despite the lack of evidence for their prognostic value, several factors were inconsistently being used as decision modifiers when selecting patients for surgical treatment.

**Supplementary Information:**

The online version contains supplementary material available at 10.1186/s40798-022-00439-6.

## Key Points

- There is no clear consensus on how to treat proximal hamstring tendon avulsions among Scandinavian surgeons.
- Several factors/decision modifiers are used when selecting patients for operative treatment despite a lack of evidence.
- Even if operative treatment of proximal hamstring tendon avulsion is a relatively uncommon procedure, half of the respondents have seen surgical complications.

## Background

Complete avulsion of the hamstring tendons (PHA) from their insertion at the ischial tuberosity is an injury typically occurring during sports participation or slip and fall accidents [1]. PHA may result in debilitating sequelae, compromising sports participation and even normal physical activity in daily life [2–4]. The outcomes following operative and non-operative PHA have only rarely been compared [1, 3], and only a few of the more than 50 published clinical studies report the results of non-operatively treated injuries [5–9]. Previous survey studies suggest that most surgeons choose treatment depending on several factors [10, 11] even though none of these factors have been studied in terms of their prognostic value. Variables of interest are the number of involved tendons, tendon retraction on MRI, patient preference, neurological symptoms and physical activity. Other unexplored factors, including patient age and body mass index (BMI), may also guide the treatment.

Ideally, orthopaedic interventions should be supported by a high level of evidence. This is especially important when the intervention, such as most surgical interventions, is associated with a risk of doing harm. When evidence is derived from retrospective observational data, there is a risk of introducing or maintaining interventions that are ineffective or even harmful.

With a probable bias in the literature favouring operative treatment, as well as limited studies reporting the outcome for non-operative treatment, it is likely that operative treatment is gaining in popularity. Crude and unpublished data from Scandinavian patient registries suggest substantial regional differences in the incidence of surgical reattachment of hamstring avulsions [12] and others have noted that clinicians are greatly overrepresented in the group of patients diagnosed with PHA [13]. We therefore suspect that there are variations in treatment policies both nationally and internationally. Understanding such variations and their underlying reasons can help to improve the implementation of guidelines as new and better evidence emerges.

This survey study primarily aims to investigate current treatment practice of PHA in the Nordic countries: Sweden, Denmark, Finland and Norway. Secondly, we aim to determine what patient- and injury-related factors influence the decision to treat patients either operatively or non-operatively and, thirdly, to collect information on perceived outcomes and complications after PHA treatment.

## Materials and Methods

### Study Population

Sweden, Norway, Denmark and Finland have comparable healthcare systems and the populations of the countries do not differ substantially in terms of ethnicity, socioeconomic status and predisposing environmental conditions. All consultant orthopaedic surgeons and trauma surgeons in Sweden were approached as potential study participants. E-mail addresses were obtained through the head of the orthopaedic surgery department at each hospital and from the Swedish Orthopaedic Society. In Norway, Denmark and Finland, the contact information of surgeons treating proximal hamstring avulsions was obtained through local knowledge of the authors and the country-specific orthopaedic society. All identified surgeons were approached using e-mail and asked to participate through an online survey. The participation was anonymous and voluntary.

The study population was selected from all responders based on the respondents’ perceived exposure to patients with PHA. Respondents that answered that they were involved in the treatment of PHA and participated in at least one PHA surgery per year were included in the study population.

### Study Design

The study is a cross-sectional survey of surgeons that are actively involved in the treatment of PHA in the Nordic countries.

### The Survey

The survey questionnaire was developed by the authors, who all are orthopaedic surgeons with experience in operative and non-operative treatment of PHA. The survey consisted of 51 items (Additional file 1: Appendix). It was designed to assess responder characteristics (9 items), the preferred current treatment practice of the surgeon as well as their units’ treatment protocols and traditions (primary outcome—13 items) and the surgeons’ opinions of patient- and injury-related factors influencing the treatment decision (secondary outcome—21 items). Furthermore, eight items investigated surgeons’ experience of complication and sequelae (tertiary outcome). Participants that felt that they never would participate in the treatment of PHA were given the option to finish the survey after providing baseline data about themselves. To circumvent the language differences and the possible bias related to forward–backward translation, the survey was provided in English. Most questions were presented in multiple-choice format.

### Survey Administration

The REDCap (Research Electronic Data Capture) database solution was used for the survey design and distribution. It is a secure, web-based software platform designed to support data capture for research studies [14]. Surgeons were approached by e-mail invitation, and study data were collected and managed using REDCap electronic data capture tools hosted at Uppsala University. Participants were able to review and change their answers through a “return to previous page” button.

Up to three reminders were sent during the subsequent six weeks if there was no response. The questionnaires were collected from April 2020 to June 2020.

### Statistical Analysis

All variables were categorical and are presented as numbers and frequencies. Percentages were calculated based on the total number of answers given for each question. To rank the surgeons’ opinion on late symptoms after PHA, we calculated a mean score for each symptom by arbitrarily giving the perceived most frequent symptom three points, the second most frequent two points and the third most frequent one point.

## Results

### Survey Reach and Response

**Fig. 1:**
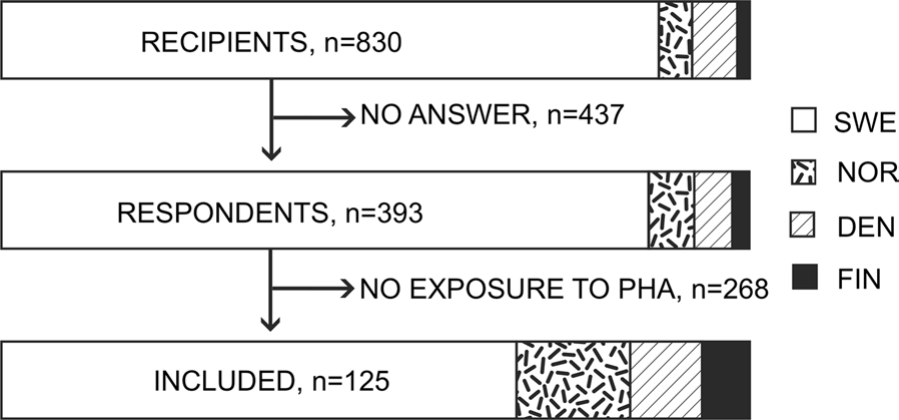
Flow-chart showing invited surgeons (*n* = 830), total number of responders (*n* = 393) and selected study population (*n* = 125). The distribution according to country is depicted with different colours

An invitation to participate was sent to 729 surgeons in Sweden, and we received 340 responses (47%). In Norway, Denmark and Finland, 37, 50 and 14 surgeons were invited to participate, respectively. We received 24, 30 and nine responses (63%). Among all the respondents in Sweden, Norway, Denmark and Finland, we identified 86, 19, 12 and eight participants with exposure to PHA patients that met our inclusion criteria. (Fig. 1).

### Demographics of Survey Respondents and their Institutions

Our study population consisted of 125 surgeons representing 38 healthcare facilities across the four countries. Most surgeons worked in non-university public healthcare facilities (95%). Similar proportions of the surgeons labelled themselves as a general orthopaedic specialist (24%), trauma specialist (33%) and sports medicine specialist (28%). However, the Danish surgeons were almost exclusively sports medicine specialists (Table 1).

**Table 1:** Study population

	Den	Nor	Fin	Swe	Total
Number of participants	12	19	8	86	125
Number of hospitals	8	16	6	31	61
Type of hospital					
Private hospital	1	0	0	5	6 (5%)
Public, non-Uni, hospital	4	14	4	43	65 (52%)
University hospital	7	5	4	38	54 (43%)
Years as orthopaedic specialist					
0–5	2	3	1	26	32 (26%)
5–10 years	3	2	5	17	27 (22%)
10–15 years	0	7	1	13	21 (17%)
> 15 years	7	6	1	29	43 (34%)
NA	0	1	0	1	2 (2%)
Subspeciality					
General orthopaedics	0	8	1	21	30 (24%)
Trauma	2	6	6	27	41 (33%)
Sports medicine	8	2	0	25	35 (28%)
Other	2	3	1	13	19 (15%)

The existence of local guidelines for the treatment of PHA was reported by 49% (60 of 123). The estimated exposure to PHA patients and the preferred diagnostic modality of the units are presented in Table 2.

**Table 2:** Unit characteristics and protocol

Local guidelines exist?	No	63	51%
	Yes	60	49%
	NA	2	
Who performs surgery at unit?	Trauma	61	49%
	Sports medicine	49	39%
	General orthopaedics	31	25%
	Referring	4	3%
	Other	9	
Number of PHA surgeons operating at unit?	0	1	1%
	1	9	7%
	2	45	37%
	3–5	61	50%
	> 5	7	6%
	NA	2	
Number of PHA patients treated at unit per annum?	0–1	14	12%
	2–5	51	43%
	5–15	46	39%
	> 15	8	7%
	NA	6	
Modality for diagnosis at unit?	Ultrasound	11	9%
	MRI	122	98%
	Clinical examination	61	49%
	Other	2	2%
General preferred treatment at unit?	Operative	16	13%
	Non-operative	13	11%
	Case dependent NA	94 2	76%

### Current Treatment Strategies and Surgeons’ View on PHA Epidemiology and Treatment

The respondents’ estimates of the gender and age distribution among PHA patients are presented in Table 3.

**Table 3:** Surgeons’ personal views of PHA epidemiology and treatment

	*n*	(%)
Gender distribution?		
More common in men	35	29
More common in women	35	29
No difference	52	43
NA	3	
Typical age of PHA patients?		
20–40 years	19	15
40–60 years	101	81
> 60 years	1	1
Evenly distributed	4	3
Is there evidence supporting surgery?		
There is little evidence for most healthy patients	22	18
There is good evidence for most healthy patients	16	13
There is evidence for a subgroup of patients	86	69
NA	1	
What should be the treatment for PHA?		
Almost always operative	8	7
Almost always non-operative	10	9
Case dependent	92	84
NA	15	

Respondents reported that most of their units took a case-by-case approach (76%) in the choice of treatment, but 24% of the units had a preference for either non-operative (11%) or operative treatment (13%) (Table 2). Sixty-nine percent (86 of 124) answered that there was good evidence for operative treatment in a subgroup of patients, while 13% (16 of 124) answered that there was good evidence supporting the operative treatment in most healthy patients and 18% (22 of 124) answered that there was no evidence for the operative treatment in most patients. When asked for their preferred treatment modality, 84% (92 of 110) said that it was case dependent, whereas 9% (10 of 110) surgeons preferred non-operative treatment and 7% (8 of 110) preferred operative treatment (Table 3).

### Factors Important for Allocation of Treatment

#### Patient Preference

If a patient favoured non-operative treatment, this was considered to be a contraindication to surgery by 48% (57 of 118) of respondents. On the other hand, 30% (35 of 118) answered that if a patient favoured operative treatment, it did not influence the treatment decision. Only 13% (15 of 118) considered such a preference to be a strong indication for surgery and 55% (65 of 118) considered it to be a weak indication (Table 4).

**Table 4:** Patient factors and clinical findings important for allocation of treatment

	*n*	(%)
Patient prefers non-operative treatment		
Strong indication for operation	4	3
Weak indication for operation	6	5
No influence	11	9
Weak indication for non-operative treatment	40	34
Contraindicates operation	57	48
NA	7	
Patient prefers operative treatment		
Strong indication for operation	15	13
Weak indication for operation	65	55
No influence	35	30
Weak indication for non-operative treatment	2	2
Contraindicates operation	1	1
NA	7	
Is age a relative contraindication for operative treatment?		
No	22	19
Yes, if age > 40	2	2
Yes, if age > 50	11	9
Yes, if age > 60	45	38
Yes, if age > 70	38	32
NA	7	
Is patient inactivity a relative contraindication for operative treatment?		
No	5	4
Yes, if extremely inactive	30	25
Yes, if sedentary lifestyle	68	58
Yes, if moderately active	9	8
Yes, if not elite athlete	6	5
NA	7	
Is patient BMI a relative contraindication for operative treatment?		
No	15	13
Yes, If BMI > 25	4	3
Yes, if BMI > 30	41	35
Yes, if BMI > 35	58	49
NA	7	
Is daily smoking a relative contraindication for operative treatment?		
No	29	25
Yes	88	75
NA	8	
Is alcohol/drug abuse a relative contraindication for operative treatment?		
No	7	6
Yes	111	94
NA	7	
No palpable proximal tendon continuity		
Strong indication for operation	31	28
Weak indication for operation	33	29
No influence	47	42
Weak indication for non-operative treatment	1	1
Contraindicates operation	0	0
NA	13	
Incapacity to actively extend the hip		
Strong indication for operation	26	23
Weak indication for operation	48	42
No influence	36	32
Weak indication for non-operative treatment	3	3
Contraindicates operation	1	1
NA	11	
Incapacity to actively flex the knee		
Strong indication for operation	35	30
Weak indication for operation	50	42
No influence	29	25
Weak indication for non-operative treatment	4	3
Contraindicates operation	0	0
NA	7	

#### Patient Characteristics and Clinical Examination

Age, BMI and the patient’s activity level were all factors that influenced treatment decisions. For example, 81% (96 of 118) of respondents considered an age above 70 to be a relative contraindication for surgery and 49% (58 of 118) considered age above 60 to be a contraindication for surgery. Similarly, 87% (103 of 118) of respondents considered severe obesity (BMI > 35) to be a relative contraindication and 38% (45 of 118) said that a BMI > 30 represented a contraindication. A sedentary lifestyle was considered a relative contraindication by 71% (83 of 118) of respondents, and only six (5%) believed that surgery should only be considered for patients with an activity level of elite athletes. Also, smoking and especially drug and alcohol abuse were considered contraindications to surgery.

Findings during clinical examination were considered to be of less importance. The inability to palpate proximal tendon continuity had either no influence or was only a weak indication for operative treatment for 71% (80 of 118) of respondents. Inability to actively flex the knee or extend the hip was considered an important indication for surgery by a subset of surgeons (30%), but the most common answer was that it either had no influence (25%) or was only a weak indication for surgery (42%) (Table 4).

#### MRI Findings and Timing of Surgery

For the participating surgeons, both the number of tendons avulsed, and the dislocation of tendons as seen by MR imaging influenced treatment decisions. Avulsion of three out of three tendons on MRI was considered a strong indication for operative treatment by 72% (85 of 118) of respondents but even the existence of two tendon avulsions was considered a strong indication for surgery by 24% (28 of 117). A retraction of 2 cm was considered a strong operative indication by 39% (45 of 116) of respondents, and if the retraction was 3 cm, an additional 35% of the respondents saw this as a strong indication for surgery.

Also, the time between injury and surgery matters in the decision-making. More than half of the surgeons felt that there was a time limit for primary operative treatment. The most commonly mentioned limits were more than four or six weeks (Table 5).

**Table 5:** MRI findings and timing of surgery

	*n*	(%)
MRI shows avulsion of two tendons		
Strong indication for surgery	28	24
Weak indication for surgery	54	46
No influence	14	12
Weak indication for non-operative treatment	19	15
Contraindicates surgery	2	2
NA	8	
MRI shows avulsion of three tendons		
Strong indication for surgery	85	72
Weak indication for surgery	26	22
No influence	6	5
Weak indication for non-operative treatment	1	1
Contraindicates surgery	0	0
NA	7	
How many cm in retraction of the tendon do YOU consider to be a strong indication for operative treatment?		
> 1 cm	3	3
> 2 cm	42	36
> 3 cm	41	35
> 4 cm	6	5
> 5 cm	14	12
No influence	10	9
NA	9	
Is there a time point where operative treatment becomes contraindicated?		
Yes, if > 2 weeks after injury	2	2
Yes, if > 4 weeks after injury	26	22
Yes, if > 6 weeks after injury	21	18
Yes, if > 10 weeks after injury	13	11
No	54	47
NA	9	

### Rehabilitation Regime and Experiences of Outcome and Complications

It was uncommon to use any bracing in the treatment of PHA but almost all respondents refer PHA patients to physiotherapy (Table 6). Return to sports for patients treated operatively was advised to be later than six months by 52% (60 of 116) of the respondents. For patients treated non-operatively, only 30% (35 of 115) of the respondents advised a return to sports later than six months and instead the most common recommendation was four to six months (Table 6).

**Table 6:** Treatment regime

	*n*	(%)
Use of orthosis for patients treated operatively?		
No	93	80
Yes	23	20
NA	9	
Use of orthosis for patients treated non-operatively?		
No	112	97
Yes	3	3
NA	10	
Referral to physiotherapist for patients treated operatively?		
No	3	3
Yes	113	97
NA	9	
Referral to physiotherapist for patients treated non-operatively?		
No	4	3
Yes	111	97
NA	10	
Return to sports for patients treated operatively?		
1–2 months	0	0
2–4 months	8	7
4–6 months	48	41
≥ 6 months	60	52
NA	9	
Return to sports for patients treated non-operatively?		
1–2 months	3	3
2–4 months	23	20
4–6 months	54	47
≥ 6 months	35	30
NA	10	

Most respondents, 62% (68 of 109), did not feel that surgically treated patients do better than non-operatively treated patients and generally felt that patients were satisfied with the treatment result, regardless of the treatment (Table 7).

**Table 7:** Treatment outcome and adverse effects

	*n*	%
In YOUR opinion: Are patients generally satisfied with the treatment and recovery regardless of whether treated non-operatively or operatively?		
Yes	68	60%
No	18	16%
No opinion	27	24%
NA	12	
Would YOU say that patients treated operatively are more satisfied than patients treated non-operatively?		
Yes	41	38%
No	68	62%
NA	16	
Have you experienced any of these complications to surgery?		
Deep infection	28	22%
Severe nerve pain	16	13%
Drop foot	3	2%
Severe knee pain	2	2%
Re-rupture of the tendon	29	23%
Thromboembolism	8	6%
No	61	49%
Average symptom score (three points for most common complaint, two points for second most common complaint and one point for third most common complaint). Total number of responders 109		
Pain when sitting		2.37
Weakness		1.70
General pain		0.85
Cramps		0.78
Numbness and/or tingling		0.71
Ischialgia		0.52
Other		0.13

About half of the respondents reported experience with serious surgical complications such as re-rupture of the tendon after surgery, deep wound infections needing debridement and severe neurogenic pain postoperatively. Pain when sitting was perceived by respondents as the most common complaint among patients with residual symptoms, followed by weakness and general pain (Table 7).

## Discussion

Our study shows a large variation between both institutional treatment traditions and individual surgeons’ opinions on how to best treat PHA. In the absence of evidence-based guidelines, many units lack local treatment guidelines, and although a majority of respondents indicated that treatment was decided on a case-by-case basis, several healthcare providers expressed a preference to treat most patients either non-operatively or operatively. Given the existing literature, it is somewhat surprising that most participants (69%) found that there was good evidence for surgical treatment in at least a subgroup of cases. In contrast, 60% of surgeons perceived patients as satisfied regardless of treatment and 62% did not believe that operated patients in general are more satisfied than non-operatively treated patients. If this perception is true, it raises the question of whether there already exists a successful, non-documented, treatment algorithm which selects the right patients for operative repair or if the treatment modality is unimportant for the outcome [8]. Nevertheless, a quicker return to pre-injury physical activity level, higher patient satisfaction and improved recovery of hamstring strength were believed to be benefits of operative treatment by many surgeons.

There are limited scientific data regarding the prognostic value of patient-related factors and preoperative findings for the functional outcome. It is also unknown what type of patient and injury surgery will be most beneficial for. Although all of the factors that were presented in our survey seemed to influence the choice of treatment modality for many of the surgeons, there was no clear consensus, with the exception that surgery is to be avoided for patients with drug or alcohol abuse and severe obesity. Delayed treatment has been shown to be associated with poor outcome [15, 16]. Accordingly, 51% of the surgeons in our survey considered delayed treatment a relative contraindication to surgery. The degree of tendon retraction has been proposed to be an important prognostic factor, and some authors suggested surgical treatment if retraction was more than 2 cm [16, 17]. The number of tendons injured was also often said to be associated with a poor outcome if treated conservatively [8], although this has not consistently been observed [7]. In our study, 39% of surgeons considered a retraction of 2 cm and 74% a tendon retraction of 3 cm to be a strong indication for operative repair. 72% answered that the existence of three avulsed tendons was an indicator for operative repair. These numbers clearly demonstrate that our respondents were aware of the suggestions proposed in the collective literature.

Previously, two studies with a similar approach have been conducted [10, 11]. Both studies identified surgeons through orthopaedic and sports medicine societies either worldwide or in the USA. Our study focused on the Nordic countries, and we aimed at identifying surgeons from all units that actively treat PHA to reach a high survey coverage. The common finding among these three studies is the apparent disparity in the management of PHA. Similar to Pasic et al. [11], we found that non-operative treatment is more common than is reflected in the published literature but the surgeons in the Nordic countries seemed to have a more positive experience of the outcome of non-operative treatment. Van der Made et al. [10] carefully examined decision modifiers and found that diminished function, in combination with imaging findings, was the strongest indicator for operative treatment. This contrasts somewhat with our finding that only a subset of respondents found that disability during a physical examination was a strong indication for surgery. In general, our study clearly showed that patient background factors, including age, obesity, alcohol consumption and physical activity level, weighed heavily on decision-making among surgeons in the Nordic countries.

Even though reattachment of the hamstrings tendons is a rare procedure, 51% of surgeons have seen surgical complications, with the most common being re-rupture of the tendon, deep wound infections needing debridement and severe neurogenic pain. Regardless of treatment, there are cases with unsatisfactory results, with pain when sitting, weakness and general pain as the most common complaints. These results are in alignment with previous surveys, demonstrating that surgeons treating PHA commonly meet patients with complications and unsatisfactory outcomes [10, 11].

Limitations of the study were the subjective nature of the survey design and that there were regional differences in how we approached surgeons. In Sweden, all surgeons with available e-mail addresses in the member database of the Swedish Orthopaedic Society and/or surgeons identified by hospital administrative offices were contacted. In the remaining countries, we directly approached surgeons who were known to treat PHA. This yielded differences in the number of responses (47% in Sweden and 63% in the other Nordic countries) and an imbalance in the number of participating surgeons per country. For this reason, our power to identify differences in answerers based on, for example, surgeon subspeciality or nationality, was inadequate. Another innate drawback of the study design is that the questions in the questionnaire were constructed with fixed answers and therefore might not reflect all aspects of the clinical diversity. Also, as we decided to include only respondents with some exposure to operative treatment of PHA, there is a risk that we missed the opinions of those that rely solely on non-operative treatment.

This study should raise awareness of the variability in practice and the inconsistent use of the different decision modifiers that are only weakly supported by clinical evidence. Understanding the variations in treatment and their underlying reasons may improve implementation of future guidelines as better evidence emerges.

## Conclusions

There was a large variability in current treatment practice and in individual opinions about the best treatment of PHA among responding surgeons. Although a majority of the respondents used multiple modifiers, patient background factors, including age, activity level and obesity, were the most consistently used modifiers. Regardless of treatment, surgeons perceived that most patients have a good clinical outcome after PHA, even though many respondents have experienced surgical complications.

## Supplementary Information

**Additional file 1: **Survey questions.

## Data Availability

The data sets used and/or analysed during the current study are available from the corresponding author on reasonable request.
