# Supplementary material for: Proximal Hamstring Tendon Avulsions: A Survey of Orthopaedic Surgeons’ Current Practices in the Nordic Countries
Source: Sports Med Open. 2022 Apr 11;8:49. doi: 10.1186/s40798-022-00439-6 (PMC9001781; doi:10.1186/s40798-022-00439-6)
Supplement: Supplementary file 1 — Additional file 1: Survey questions. [file 40798_2022_439_MOESM1_ESM.pdf]

The PHACT survey study - a survey about complete proximal hamstring avulsions

What is the name of YOUR workplace?	__________________________________
I work in:	◯ Private hospital<br>◯ Public hospital<br>◯ University hospital<br>◯ Other
Please specify "Other":	__________________________________
Are you a specialist in orthopedic surgery?	◯ Yes<br>◯ No
For how long have you practiced as an orthopedic specialist?	◯ 0-5 years<br>◯ 5-10 years<br>◯ 10-15 years<br>◯ more than 15 years
Subspeciality?	◯ General orthopedics<br>◯ Trauma<br>◯ Sportsmedicine<br>◯ Other
Please specify "Other":	__________________________________
If YOU are unlikely to see or treat patients with proximal hamstring avulsions you can exit the survey by ticking the appropriate box:	◯ Keep going<br>◯ I am not involved in the treatment of proximal hamstring avulsions
How many patients with a proximal hamstring avulsion do YOU see during a year?	◯ 0-1<br>◯ 2-5<br>◯ 5-10<br>◯ >10
How many patients with proximal hamstring avulsions do YOU operate or assist on during a year?	◯ I never operate on these injuries<br>◯ 0-1<br>◯ 2-5<br>◯ 5-10<br>◯ >10
What is YOUR current opinion on the evidence guiding the treatment of proximal hamstring avulsions?	◯ There is little/no evidence for the operative treatment in most healthy patients<br>◯ There is good evidence supporting the operative treatment in most healthy patients<br>◯ Operative treatment is an option in a subgroup of patients
Would YOU consider the injury to be more common in men or women?	◯ More common in men<br>◯ More common in women<br>◯ No difference

What would YOU say is a typical age for a patient with a proximal hamstring avulsion?	◯ 20-40 years<br>◯ 40-60 years<br>◯ >60 years<br>◯ The injury is evenly distributed among different age groups
Does your UNIT have local guidelines for the management of proximal hamstring avulsions?	◯ Yes<br>◯ No
Who performs the operation on a proximal hamstring avulsion? (multiple answers possible)	☐ A specialist from the trauma section<br>☐ A specialist in sports medicine<br>☐ A general orthopedic surgeon<br>☐ We don´t perform the surgery at our clinic<br>☐ Referral to other centre<br>☐ Other
Please specify other:	__________________________________
How many surgeons performs reattachments of hamstring avulsions in your UNIT?	◯ 0<br>◯ 1<br>◯ 2<br>◯ 3 - 5<br>◯ >5
How many proximal hamstring avulsion patients are treated in your UNIT per year?	◯ 0-1<br>◯ 2-5<br>◯ 5-15<br>◯ >15
What is the preferred modality used to diagnose a suspected proximal hamstring avulsion at your UNIT? (multiple answers possible)	☐ Ultrasound<br>☐ MRI<br>☐ Clinical examination<br>☐ Other
Please specify "Other":	__________________________________
What would you say is the preferred treatment for proximal hamstring avulsions at your UNIT in general?	◯ Almost always operative<br>◯ Almost always non-operative<br>◯ It depends on the individual case
What in YOUR opinion should be the treatment for proximal hamstring avulsions?	◯ Almost always operative<br>◯ Almost always non-operative<br>◯ It depends on the individual case

How do the following factors affect YOUR decision to treat a proximal hamstring avulsion?

	Strong indication for operative treatment	Weak indication for operative treatment	No influence on the decision	Weak indication for non-operative treatment	Contradicts operative treatment

No palpable proximal tendon continuity	○	○	○	○	○
MRI shows avulsion of 2 out of 3 tendons	○	○	○	○	○
MRI shows avulsion of 3 out of 3 tendons	○	○	○	○	○
Incapacity to actively flex the knee	○	○	○	○	○
Incapacity to actively extend the hip	○	○	○	○	○
Patient prefers operative treatment	○	○	○	○	○
Patient prefers non-operative treatment	○	○	○	○	○

Comments? ______________________________

How many cm in retraction of the tendon do YOU consider to be a strong indication for operative treatment?

○ > 1 cm ○ > 2 cm
○ > 3 cm ○ > 4 cm
○ > 5 cm ○ No influence on decision of operative treatment

Do you consider the level of patient inactivity to be a relative contraindication for operative treatment?

- ○ No
- ○ Yes, if extremely inactive
- ○ Yes, if sedentary lifestyle (office worker getting little or no exercise)
- ○ Yes, if moderately active (construction worker or person exercising weekly)
- ○ Yes, if not elite athlete

Is the age of the patient a relative contraindication for operative treatment?

- ○ No
- ○ Yes, if age>40
- ○ Yes, if age >50
- ○ Yes, if age >60
- ○ Yes, if age >70

Is patient BMI a relative contraindication for operative treatment?

- ○ No
- ○ Yes, If BMI >25
- ○ Yes, if BMI >30
- ○ Yes, if BMI >35

Is the fact that the patient smokes daily a relative contradiction for operative treatment?

- ○ No
- ○ Yes

Is the fact that the patient has an alcohol/drug abuse a relative contradiction for operative treatment?

- ○ No
- ○ Yes

QUESTIONS REGARDING THE OPERATIVE TREATMENT of PROXIMAL HAMSTRING AVULSIONS

Are these, in YOUR opinion, BENEFITS of OPERATIVE treatment?

	Yes	No
Quicker return to pre-injury physical activity level	◯	◯
Less risk of re-rupture	◯	◯
Higher patient satisfaction	◯	◯
Improved recovery of hamstrings strength	◯	◯

Comments?

____

According to YOU; is there a time point where OPERATIVE treatment becomes contraindicated?

◯ Yes, if > 2 weeks after injury
◯ Yes, if > 4 weeks after injury
◯ Yes, if > 6 weeks after injury
◯ Yes, if >10 weeks after injury
◯ No

Do YOU recommend a brace orthosis for patients treated OPERATIVELY?

◯ Yes
◯ No

What is YOUR advice concerning period of return to sports for patients treated OPERATIVELY?

◯ 1- 2 months
◯ 2- 4 months
◯ 4- 6 months
◯ ≥ 6 months

Do YOU refer the patient to a physiotherapist if treated OPERATIVELY?

◯ Yes
◯ No

QUESTIONS REGARDING THE NON-OPERATIVE TREATMENT of PROXIMAL HAMSTRING AVULSIONS

Are these, in your opinion, BENEFITS of NON-OPERATIVE treatment?

	Yes	No
Quicker return to pre-injury physical level	◯	◯
Less complications	◯	◯
Higher patient satisfaction	◯	◯

Comments? __________________________________

Do YOU recommend a brace orthosis for patients treated NON-OPERATIVELY?
- ◯ Yes
- ◯ No

What is YOUR advice concerning period of return to sports for patients treated NON-OPERATIVELY?
- ◯ 1- 2 months
- ◯ 2- 4 months
- ◯ 3- 6 months
- ◯ ≥ 6 months

Do you refer the patient to a physiotherapist if treated NON-OPERATIVELY?
- ◯ Yes
- ◯ No

In YOUR opinion: Are patients generally satisfied with the treatment and recovery regardless if treated non-operatively or operatively?
- ◯ Yes
- ◯ No
- ◯ No opinion

Would YOU say that patients treated operatively are more satisfied than patients treated non-operatively?
- ◯ Yes
- ◯ No

Have YOU experienced any of these complications to surgery? (multiple answers possible)
- ☐ Wound infection needing debridement
- ☐ Severe nerve pain
- ☐ Drop foot
- ☐ Severe knee pain
- ☐ Re-rupture of the tendon
- ☐ Thromboemboli
- ☐ No experience of these complications

In cases where the outcome is dissatisfactory, what in YOUR experience, is the MOST common complaint?
- ◯ Weakness
- ◯ Instability of the knee
- ◯ Pain when sitting
- ◯ Ischialgia
- ◯ Numbness and/or tingeling
- ◯ General pain
- ◯ Cramps
- ◯ Other

Please specify "Other": __________________________________

In cases where the outcome is dissatisfactory, what in your experience, is the SECOND MOST common complaint?	◯ Weakness<br>◯ Instability of the knee<br>◯ Pain when sitting<br>◯ Ischialgia<br>◯ Numbness and/or tingeling<br>◯ General pain<br>◯ Cramps<br>◯ Other
Please specify "Other":	__________________________________
In cases where the outcome is dissatisfactory, what in your experience, is the THIRD MOST common complaint?	◯ Weakness<br>◯ Instability of the knee<br>◯ Pain when sitting<br>◯ Ischialgia<br>◯ Numbness and/or tingeling<br>◯ General pain<br>◯ Cramps<br>◯ Other
Please specify "Other":	__________________________________
Please feel free to submit comments and thoughts here:	__________________________________

Tack för att du tog dig tid och svarade på frågorna.

Ha en fortsatt trevlig dag!

Thank you for taking the time to complete this survey.

Have a nice day!
